# Sociodemographic factors in Arab children with Autism Spectrum Disorders

**Published:** 2012-11-26

**Authors:** Mostafa Amr, WaleedAl Bu Ali, Hatem Hablas, Dahoud Raddad, Fatma El-Mehesh, Abdel-Hady El-Gilany, Hemdan Al-Shamy

**Affiliations:** 1 Assistant Prof. of Psychiatry, College of Medicine, Mansoura University, Egypt; 2 Assistant Prof. of Pediatrics, College of Medicine in King Faisal University, Saudi Arabia; 3 Consultant of Psychiatry, National center for Mental Health Hospital, Amman, Jordan; 4 Assistant Prof. of Pediatrics, College of Medicine in Al-Azhar University, Egypt; 5 Consultant of Psychiatry, Psychiatric Hospital in Al-Ahsa, Saudi Arabia; 6 Professor of Community Medicine, College of Medicine, Mansoura, Egypt; 7 Lecturer of Psychology, College of education, King Faisal University, Saudi Arabia

**Keywords:** Autistic spectrum disorders, maladaptive behavior, autistic symptoms, socio-demographic variables

## Abstract

**Introduction:**

There is a critical gap in Autistic Spectrum Disorders (ASD) research with respect to manifestations of the condition in developing countries This study examined the influence of sociodemographic variables on the severity of autistic symptoms and behavioral profile in Arab children.

**Methods:**

The total study sample comprised of 60 Arab children (38 boys and 22 girls) from three Arab countries (22 Jordanians, 19 Saudis and 19 Egyptians). The diagnosis of Autism Spectrum Disorders (ASD) was based on DSM-IV criteria supplemented by direct observation according to the Indian Scale for Assessment of Autism (ISAA) and assessment of Intelligent Quotient (IQ). Finally, parents rated their child on the Achenbach Child Behavior Checklist (CBCL).

**Results:**

It was found that the housewives and Saudi parents described more autistic symptoms and externalizing behavior problems. A significant negative correlation was found between IQ and each of ISAA, CBCL Internalizing and Externalizing problems scores.

**Conclusion:**

The study concluded that the clinical presentation of ASD may be shaped by cultural factors that are likely to help to formulate specific diagnosis and intervention techniques in Arab children with ASD.

## Introduction

Autism Spectrum Disorders (ASD) are referred to as Pervasive Developmental Disorders (PDD) in the Diagnostic and Statistical Manual of Mental Disorders [1] (American Psychiatric Association, 2000) and the ICD-10 Classification of Mental and Behavioral Disorders [2]. The concept of ASD includes autistic disorder, Asperger syndrome, and PDD not otherwise specified (PDDNOS), which includes atypical autism. ASD is a disorder of the developing brain, mainly presenting with a distinct pattern of social deficits, communication impairment, and rigid ritualistic interests [1]. These symptoms often manifest within the first three years of life, and persist throughout life. Associated with mental retardation and seizure disorder in a significant number of cases, it is influenced mainly by genetic factors: 90% of the variance is said to be cause by genetic factors [3]. Recent reports have suggested that the prevalence of autism and related spectrum disorders (ASDs) is substantially higher than previously recognized. The prevalence of autistic disorders was 38. 9 per 10,000 and that of other ASDs was 77. 2 per 10,000, making the total prevalence of all ASDs 116. 1 per 10,000 [4, 5]. With regard to the gender difference, recent studies reported that ASD occurs more commonly in males than females with a ratio of 3 or 4:1 [6]. The sex ratio moves closer to 1:1 for the children with autism who are profoundly retarded [7, 8]. Since the 1970s, there has been a dramatic rise in the number of reports documenting increasing rates of ASD cases, especially in western countries (Lenoir et al., 2009) [9]Very few reports have been published about the occurrence of ASD in Arab countries, especially those of the Middle East [10]. One recent report estimated an overall prevalence of 1. 4 ASD cases per 10,000 children in Oman [11]. Many factors may contribute to the substantial under-diagnosis of ASD cases in developing countries. One of the factors is the difficulty in obtaining a diagnosis, as the pediatricians are often inexperienced in the diagnosis and management of these disorders. In general there are few psychiatrists specializing in childhood problems [12]. The lack of awareness among parents regarding ASD, including a failure to recognize symptoms and seek diagnosis and treatment is also likely to be a factor, especially in cases of children with mild forms of the disorder [11].

In this study, we attempt to examine the problem in three Arab countries: Egypt, Saudi Arabia and Jordan. These three countries share a number of similarities. Foremost among these are the language and religion. These nations also share mutual good relations and there is generally a mutual respect between the people of the three countries. There are however significant social, cultural and economic differences. The national income of Saudi Arabia is much higher than that of Egypt and Jordan, while the rate of social change is faster in Egypt and Jordan than that of Saudi Arabia. In comparison to the Saudis, Egyptians and Jordanians are less conservative as their values are more related to the western societies and are not shaped by the norms of the tribal society [13].

The aim of this pilot study was to clarify the relationship of sociodemographic variables and ASD. Specifically; we wished to investigate whether these factors explained more of the variation in autistic symptoms and behavior profile among children with ASD. We hypothesized that; consistent with the previous studies, those younger male children with lower parental educational, occupational and economic status would evidence more autistic symptoms and behavior patterns. Such undertaking is likely to help to formulate diagnosis and treatment techniques, as well as provide a basis for assessment of the magnitude of ASD in Arab world.

## Methods

### Participants

We examined 60 children (38 boys and 22 girls) with a diagnosis of ASD that were recruited from three centers: Center for Early Diagnosis of Children′s Disabilities (EDCD), Amman, Jordan (22 children), child psychiatry settings in Mansoura University Hospital(MUH), Egypt (19 children) and Al-Ahsa psychiatric Hospital, Saudi Arabia (19 children) (Table 1). This is the same population studied in recently published studies on sex differences and co-morbid psychiatric conditions in Arab children with ASD [14, 15].

**Table 1:** Sociodemographic features of studied autism cases according to country

	Egypt [19]	Saudi Arabia [19]	Jordan [22]
**Age at diagnosis** (mean ± SD)	8.6±2.0	8.1±1.8	7.9±2.2
**Sex**			
Boys	9(47.4)	15(78.9)	13(59.1)
Girls	10(52.6)	4(21.1)	9(40.9)
**Father education**			
Above secondary	5(26.3)	6(31.6)	8(36.6)
≤ Secondary	14(73.7)	13(68.4)	14(63.4)
**Father occupation#**			
Professional or clerical worker	4 (21)	5(26.3)	6(30.0)
Farmers or manual worker	13(76.5)	12(70.6)	14(70.0)
**Mother education**			
Above secondary	3(15.8)	5(26.3)	5(22.7)
≤ Secondary	16(88.9)	14(73.7)	17(81.0)
**Mother occupation**			
# House wises	12(66.7)	12(63.2)	14(66.7)
Working outside home	6(33.3)	7(36.8)	7(33.3)
**Income**			
Satisfactory*	12(63.2)	6(31.6)	17(77.3)
Unsatisfactory	7(36.8)	13(68.4)	5(22.7)
**Residence**			
Rural	7(36.8)	9(47.4)	3(13.6)
Urban	12(63.2)	10(52.6)	19(86.4)
**Family size**			
≤ 4 members	9(47.4)	15(78.9)	13(59.1)
>4 members	10(52.6)	4(21.1)	9(40.9)

# Six deceased fathers and two mothers were excluded from analysis

* Satisfactory income means sufficient enough to satisfy the living needs of the family (e.g., accommodation, food, transportation, clothes, costs of education for children and health care) without any in-debt.

To be eligible for the study, patients had to present with the typical triad of symptoms of autism: social deficits; communication impairment; and rigid ritualistic interests. Diagnosis of autistic spectrum disorders (also referred to as pervasive developmental disorders) was based on the DSM-IV-TR criteria [1] and included the categories of autistic disorder; Asperger's disorder and Pervasive Developmental Disorder Not Otherwise Specified (PDDNOS). Patients were diagnosed by child psychiatrists in each country after direct interviews with the child and the parents. Autistic disorder was diagnosed in 34 boys and 21 girls, the PDDNOS in four boys and one girl. None of the participants were diagnosed with Asperger′s disorder. The clinical diagnosis was corroborated using the Arabic version of the Indian Scale for Assessment of Autism (ISAA) [16]. During the diagnostic work-up, the Arabic version of the CBCL 4-18 was given to the study participants’ parents. Given the range in age and level of ability, various intelligence tests were used to assess intellectual functioning. In 60 patients, IQ was assessed using the Arabic version of the Wechsler Intelligence Scale for Children (WISC) (n= 22) and the Stanford Binet Intelligence Test (n= 33). Clinical assessment was used to define intellectual disabilities in five participants. In all, 43 patients were taking psychotropic medications for co-morbid disorders. The study was approved by the local medical ethics committee in the three countries and an informed consent from the parents of each of the children had been obtained.

### Tools

**A semi-structured questionnaire:** The questionnaire included the various demographic and academic characteristics including age, gender, educational status of the child, parental educational and occupational status,family size, income and residence.

**Indian Scale for Assessment of Autism (ISAA):** The ISAA was commissioned by the National Institute for the Mentally Handicapped [16] as a suitable tool for identification and rating the severity of autism in developing countries as opposed to the present tests that have mostly western parameters, like the Childhood Autism Rating Scale (CARS). Children were rated according to the ISAA, based on behavioral observation and interaction with the examiner and parents. The ISAA evaluation was completed by an independent, qualified child psychiatrist, blinded to the DSM-IV-TR diagnosis. The scale has 40 items based on DSM-IV-TR criteria, divided into six domains: social relationship and reciprocity; emotional responsiveness; speech, language and communication; behaviour problems; sensory aspects and cognitive problems. Participants’ behavior was rated on a five point scale (rarely, sometimes, frequently, mostly and always). According to the ISAA manual, autism is defined by a score of 70 points. Total scores of 70 to 106 indicates mild autism, 107-153 moderate autism, and scores of 153 and above indicates severe autism. The cut-off point had a sensitivity of 94. 3% and a specificity of 92. 0%. The Cronbach's coefficient alpha of internal consistency was 0. 97 and inter-rater reliability coefficient was 0. 83 (p14].

**The Child Behavior Checklist (CBCL):** The CBCL is a standardized instrument for the assessment of a child's behavioral problems. It is suitable for children aged from 4 to 16 years and can be completed in 15 - 17 min by the parents. The instrument is easily applied, and there is a lot of data showing its high test-retest reliability and discriminate validity, which also applies to the Arabic version [17]. The items of the CBCL are divided into eight domains, each of which takes different aspects of behavior into account: withdrawn, somatic complaints, anxious/depressed state, social problems, thought problems, attention problems, delinquent behavior, and aggressive behavior. Most of the eight domains within the CBCL can be subdivided into two subscales: internalizing problems and externalizing problems. These two subscales reflect a distinction between inhibited/anxious behavior (internalizing) on the one side and aggressive, antisocial, behavior (externalizing) on the other side. The internalizing subscale is a summation of withdrawn, somatic, complaints, and anxious/depressed state. The externalizing subscale is comprised of aggressive behavior and delinquent behavior [18]. Norms for these standardized questionnaires in the Arab countries [19, 20] have not yet been established. According to the American norms, standardized T scores of 65-69 fall into the "borderline clinical" range, while scores of 70 and above can be regarded as clinically significant [18].

### Procedures

Parents, mainly mothers, were interviewed by three experienced child psychiatrists employing a clinical interview according to DSM-IV-TR criteria for ASD in the first visit. The second visit involved IQ assessment of the child while ISAA and CBCL form was self-completed by one of the parents.

### Statistical analysis

Data was analyzed using SPSS program version 16. Descriptive statistics were presented as mean ± SD for quantitative variables and as number and percent for qualitative variables. Unpaired student's t-test was used for two group comparison and ANOVA (F-test) with test of least significant difference were used for the three groups′ comparison.

## Results

Table 1 shows the socio-demographic characteristics of the total sample. Mean age of the total sample was 8. 2 ± 2 with a range from 4 to 11 years. Boys represented the majority of cases in Saudi Arabia. Most of the children diagnosed with autism belonged to families of low socioeconomic standards with unsatisfactory income, especially in Egypt and Jordan, along with a lower paternal education level. Most of affected children were from urban areas probably because they had a better access to the three psychiatry facilities involved in this study in the three countries, especially in Egypt and Jordan. The total IQ mean scores of the included children was 60. 93 ± 20. 86 and approximately two-thirds of the sample (60%) had mental retardation. There were no significant difference in the IQ scores in relation to country of residence (Kruskal-Wallis, P = 0. 685).

Table 2 showed that housewives reported that their children displayed more autistic symptoms and externalizing problems than the working mothers Also, Saudi parents scored their children highest on CBCL externalizing while the Jordanians scored the lowest.

**Table 2:** Variation of IQ, ISAA, CBCL Internalizing, and externalizing problems scores according to different sociodemographic factors

Independent variables		ISAA		Internalizing		Externalizing	
		Mean ± SD	t	Mean ± SD	t	Mean ± SD	t
**Sex**	Male	109.6±32.0	0.04	67.6±7.9	0.6	67.4±6.8	1.3
	Female	109.3±27.6		66.1±6.0		64.9±8.2	
**Family size**	≤ 4members	115.6±33.0	0.51	67.5±8.2	0.18	67.2±6.8	0.28
	>4 members	105.1±27.6		66.8±6.6		65.8±7.8	
**Father education**	Above secondary school	110.3±30.6	0.6	67.2±6.5	0.8	66.6±7.3	0.1
	≤ secondary school	105.2±19.6		65.9±3.2		66.4±5.6	
**Mother education**	Above secondary school	108.3±29.6	0.03	66.9±7.8	0.7		0.2
	≤ secondary school	108.0±18.7		68.7±4.6		66.3±7.7	
**Father occupation**	Professional	104.6±20.1	0.7	65.9±3.3	0.7	66.1±5.7	0.8
	Manual worker	110.4±30.2		67.1±6.4		66.4±7.2	
**Mother occupation**	Housewives	116.1±28.5	0.005*	67.9±7.8	1.0	68.6±6.1	0.000*
	Working outside home	93.4±27.3		66.0±6.3		61.6±7.6	
**Income**	Unsatisfactory	108.1±33.3	0.4	66.1±7.6	1.3	65.4±8.2	1.2
	Satisfactory	111.4±25.5		68.5±6.5		67.8±5.9	
**Residence**	Rural	105.7±29.9	1.4	66.1±5.9	1.5	66.1±7.4	0.5
	Urban	117.6±29.8		69.2±9.4		67.2±7.4	
**Country**	Egypt	110.8±31.0	F=0.6	68.3±8.4	F=0.2	65.6±7.6	F=0.01
	Saudi Arabia	110.2±23.0		68.7±7.4		70.1±4.7*	
	Jordan	107.7±35.6		64.7±5.5		64.0±8.0*	

* Significant difference between the corresponding groups, least significant difference test; ISAA: Indian Scale for Assessment of Autism; CBCL: Achenbach Child Behavior Checklist

Table 3 showed a significant positive correlation between ISAA and CBCL Internalising scores with the CBCL Externalising score while IQ has a negative correlation with ISAA, CBCL Internalising and Externalising scores.

**Table 3:** Inter-Correlation between IQ, ISAA, CBCL Internalizing, externalizing problems scores

	IQ	ISSA	Internalizing	Externalizing
**ISSA**	-0.87***			
**Internalizing**	-0.26*	0.23		
**Externalizing**	-0.51**	0.63***	0.34**	

* significant correlation at P ≤0.05

** significant correlation at P ≤0.01

*** significant correlation at P ≤0.001

ISAA: Indian Scale for Assessment of Autism; CBCL: Achenbach Child Behavior Checklist

## Discussion

Our study has investigated the characteristics of ISAA and CBCL in three groups of young children with ASD. It was noted that there was a significant correlation with working status of the mother, country of origin and intellectual function on the ISAA and CBCL. Housewives reported that their children displayed more autistic symptoms and externalizing problems than the working mothers. Parents from Saudi Arabia described more externalizing problems in their children as compared to parents from Jordan. A significant negative correlation was found between IQ, ISAA and CBCL scores whereas there is a significant positive correlation between ISAA and CBCL Externalizing problems scores.

Gender was not significantly correlated with ISAA and CBCL scoring. This result contradicts previous findings [21] in a cohort study that included 118 children with autism followed into adolescence which reported more significant social impairment among females It is also ascertained that girls with AD have a slightly more severe presentation than boys although gender differences are limited. However, other studies have not found gender differences in language level, unusual verbal behaviors, or the level of repetitive behaviors [22, 23]. However, the small sample size and the odd gender split of the current study,many more females than would be expected based on the typical gender ratios in previous studies [6] should be acknowledged.

It is difficult to determine whether this finding reflects differences in ISAA and CBCL because the sample was recruited from three Arabic countries only and therefore may not be representative of the whole autistic population in Arab countries and also may not be comparable to the study populations of previous studies. Furthermore, past research has examined four individual characteristics in addition to gender that may be important correlates of the level of ISAA and CBCL: Age, a co-morbid diagnosis of mental retardation, education, socioeconomic level and race [24].

Our findings that children from Saudi Arabia had more externalizing problem CBCL scores compared to those from Jordan, support the previous research in Gulf region from United Arab Emirates and Saudi Arabia [25, 26].

They suggested that externalizing symptoms (e. g. hyperactivity and impulsivity) were more easily detected than the internalizing symptoms among Gulf children with ASD because of the additional burden to the children's caregivers. It was hypothesized that parenting styles vary across Arab countries, with parenting style in traditional countries such as Yemen and Saudi Arabia tending to be more authoritarian than parenting styles in modern countries such as Lebanon and Jordan [27]. Children of authoritarian parents manifest more behavior problems than youth having authoritative or permissive parents [28]. However, the Saudi parents might be more sensitive regarding aggressive/dissocial behavior and tend to overestimate such behaviors.

Few researchers have attempted a comparative approach or explicitly addressed socio-demographic risk factors in ASD. Early descriptive studies conducted among individuals with autism found a preponderance of parents from high social class backgrounds, as defined by their occupation, education, or intellect level [29, 30].

Recent investigations have not observed any association between higher social class and autism [31–34]. The study showed that housewives tended to more frequently report that their children displayed more autistic symptoms and externalizing problems compared to the working mothers. It has been shown that children with ASD whose mothers were working might have more financial resources, better understanding about ASD and access to therapeutic interventions and enrolment in regular or special education schools in Egypt [35]. Several limitations of our study should be considered. The small sample size with predominance of children with autistic disorder and relatively few with PDD NOS compared to the latest estimates on relative sizes of ASD subgroups [36]. Further, there were many children with low intellectual level. Another limitation was the odd gender split (many more females than would be expected in previous studies particularly from Egypt and Jordan) Thus, our sample, though from several countries, cannot be seen as representative for the entire population of Arab children with ASD, and the generalizability of our results is limited. In addition, information from parents concerning the child′s medical and developmental history is absent, thus the extent to which children had co-morbid syndromes (e. g., Fragile X syndrome) or medical problems that may have impacted their autistic symptoms, or coexisting behavior problems is not known.

The major challenge in our paper is dissecting the difference between inherent, biologically determined characteristics of children with ASD, and cultural influences on the manner in which autism is expressed either as judged by parents or as a result of parenting [25–27, 37]. We note that there are markedly different parenting patterns for children with ASD as well as very likely large differences in expectations. This raises the possibility that the scores on the two scales reflect deviation from cultural expectations for boys and girls as much, or perhaps even more, than inherent differences in expression of autism characteristics. Previous studies have found that intensive, sustained special education programs and behavior therapy early in life can help children with ASD to acquire self-care, social, and job skills [38], and often improve functioning and decrease symptom severity and maladaptive behaviors such as aggression, defiance and inattention [39]. These findings suggest that that children with autism with working mothers are more likely to be enrolled in special education programs.

## Conclusion

Autism is biological disorder. It exhibits the same core deficits in all cultures. However, the clinical presentation of ASD may be shaped by culturally determined factors and needs more elaborate/different intervention programs. The cultural context may significantly influence the parental expectation and family concerns about managing the problem. Future studies should include a comparison group, such as children with mild intellectual disability but without autism. This may further clarify the parental expectation differences for both boys and girls in other groups with intellectual and developmental disabilities compared with autism as well. Also, because the co-occurring behavior problems were gleaned from parent-reports in the CBCL, the potential effects of parental biases must be considered. Additional assessment by structured clinical interviews and observational measures would have been desirable to provide a more comprehensive view of the participant's problems. We need to include cultural, behavioral and educational management strategies in any comprehensive intervention program for young Arab children and adolescents with ASD.

## Competing interests

The authors declare no competing interests.

## Authors’ contributions

MA designed the study, supervised the data collection and wrote the paper. WAA, HR, DR, FEM collected the data and assisted with writing the article. AHG and HAS were responsible for the statistical design of the study and for carrying out the statistical analysis. All the authors have read and approved the final version of the manuscript.
